# Correlation between Prenatal Exposure to Polybrominated Diphenyl Ethers (PBDEs) and Infant Birth Outcomes: A Meta-Analysis and an Experimental Study

**DOI:** 10.3390/ijerph14030268

**Published:** 2017-03-07

**Authors:** Xuemin Zhao, Shiqiao Peng, Yang Xiang, Yali Yang, Jing Li, Zhongyan Shan, Weiping Teng

**Affiliations:** Department of Endocrinology and Metabolism, Institute of Endocrinology, The First Affiliated Hospital of China Medical University, Liaoning Provincial Key Laboratory of Endocrine Diseases, Shenyang 110001, China; zhaoxuemincmu@126.com (X.Z.); clearling405@163.com (S.P.); xiangyang0430@outlook.com (Y.X.); 13324086880@163.com (Y.Y.); shanzhongyan@medmail.com.cn (Z.S.); twp@vip.163.com (W.T.)

**Keywords:** polybrominated diphenyl ethers, birth outcomes, birth weight, head circumference

## Abstract

Only a few epidemiological studies have focused on the correlation between prenatal exposure to polybrominated diphenyl ethers (PBDEs) and infant birth outcomes (IBO), and the results of these epidemiological studies are contradictory. The objective of this study was to assess the correlation between prenatal exposure to PBDEs (PEP) and IBO (i.e., birth weight) in an analysis of epidemiological studies and an experimental animals study. We searched databases of the medical literature (PubMed, Excerpta Medica Database, Cochrane Library, and China National Knowledge Infrastructure) for articles and pooled the results of the included epidemiological studies. In parallel, birth outcomes (i.e., birth weights of pups) were collected from female Sprague-Dawley (SD) rats exposed to decabromodiphenyl ether (DecaBDE) in the diet from five weeks of age to delivery. A significant negative relationship was found between human PBDE burden and birth weight in the analysis of seven epidemiological studies based on a random-effects model (β = −50.598; 95% confidence interval (CI) −95.914, −5.282; *I*^2^ = 11.8%; *p* = 0.029). In the experimental animal study, a significant decrease in birth weight in the DecaBDE-treated group was also observed (5.26 ± 0.39 vs. 5.8 ± 0.58, *p* = 0.0132). The results of our study contribute to increasing evidence suggesting that PEP adversely impacts IBO, especially birth weight.

## 1. Introduction

As additive flame retardants, polybrominated diphenyl ethers (PBDEs) have been added to a variety of consumer and commercial products, such as furniture, electrical equipment, textiles, household appliances, and other materials, for more than four decades worldwide. PBDEs, which consist of 209 congeners, are likely to leach out of such products in dust during their lifetime [1,2]. Due to the ubiquity and properties of PBDEs, including bioaccumulation, lipophilicity, and persistence [3], these compounds have been found in adipose tissues, milk in females, and human serum in almost all individuals [4,5,6]. Several studies have suggested that the adverse effects of PBDE exposure include obesity, endocrine disruption, neurotoxicity, learning disabilities, and reproductive toxicity [7,8,9,10]. Several studies [11,12] found that an increasing burden of PBDEs was negatively related to birth weight. However, no relationship between PBDE exposure and birth weight was found in other epidemiological studies [13,14,15]. These conflicting results were also observed in experimental animal studies. One study performed by Du et al. revealed that prenatal exposure to decabromodiphenyl ether (DecaBDE) reduced the birth weight of newborn Sprague-Dawley (SD) rats [16]. Another animal study did not find a difference in the birth weight of newborn SD rats between the DecaBDE-treated group and the control group [17]. Currently, there is no convincing conclusion regarding this relationship. The primary aim of our study was to comprehensively evaluate the correlation between prenatal exposure to PBDEs (PEP) and birth weight in an analysis of epidemiological studies and an experimental animal study.

## 2. Materials and Methods

### 2.1. Analysis of the Results of Epidemiological Studies

#### 2.1.1. Search Strategy

The protocol for our analysis was registered in PROSPERO with the number CRD42016047864. Medical literature databases, including the China National Knowledge Infrastructure (CNKI), Cochrane Library, PubMed, and Excerpta Medica Database (Embase), were searched from June 1980 to June 2016 to identify studies analyzing the correlation between PBDE levels and infant birth weight. To identify potential studies, we also examined the reference lists of the obtained epidemiological studies. A total of 102 potential articles were identified using the following keywords: polybrominated diphenyl ethers, PBDE, birth weight, birth outcome, etc. Then, the titles and abstracts of these studies were independently screened by three authors (X.Z., S.P., Y.X.) to determine the relevance of the study to the aim of our research.

#### 2.1.2. Selection Criteria

Thirteen relevant studies met the following inclusion criteria: (a) epidemiological studies; (b) studies designed to investigate the relationship between PEP (ng/g lipids) and infant birth outcomes (IBO); and (c) studies written in the English language. These potential studies were subsequently examined by full-text review. Studies were excluded if the following criteria were fulfilled: (a) regression coefficients and the 95% confidence interval (CI) or standard error (SE) in the studies were unavailable; and (b) studies were reviews, conference abstracts, books, or reports.

#### 2.1.3. Data Extraction and Quality Assessment

After identifying relevant human studies, three researchers (X.Z., Y.X., and J.L.) independently extracted the following general information and data (Table 1): author name, country, gender, publication year, number of participants, effect size, and units. The methodological quality of all five included articles [12,14,15,18,19] was assessed independently by three researchers according to a cross-sectional assessment tool [20,21]. We considered the five studies to be of high quality.

#### 2.1.4. Data Analysis

β values with the corresponding 95% CIs or SEs were acquired from each study. The calculation of pooled β values was conducted using Stata SE 12.0 (StataCorp, College Station, TX, USA). The random-effects model was used to perform all analyses [22]. The statistical heterogeneity of β values between different studies was assessed using the chi-square-based Q test and *I*^2^ test, with *p* < 0.05 indicating statistical significance [23,24]. Sensitivity analysis was subsequently performed by contrasting the results between two models (fixed-effects and random-effects models). Egger’s test [25] and Begg’s test [26] were used to assess the publication bias of our study.

### 2.2. Animal Study Design and Samples

#### 2.2.1. Animals and Housing

The protocol of our animal study was evaluated and approved by the Ethics Committee of China Medical University (project identification code, 15028R; date of approval, 27 July 2015). Forty-eight female Sprague-Dawley rats (one week after weaning) weighing 85 ± 10 g were obtained from Beijing Wei Tong Li Hua (Vital River) Laboratories (Beijing, China). All 48 SD rats were housed on a twelve-hour light: twelve-hour dark cycle (6:00 a.m.–6:00 p.m.) in the Animal Experimental Centre of China Medical University, with free access to animal food and tap water ad libitum (specific pathogen-free conditions).

#### 2.2.2. Experimental Design

**Figure 1:**
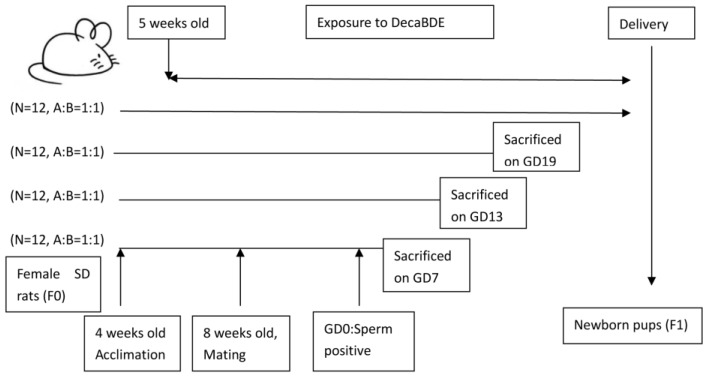
Experimental design of the animal study. Forty-eight Sprague-Dawley (SD) rats were given 1000 mg bromodiphenyl ether (DecaBDE)/kg birth weight (bw) per day from 5 weeks of age to delivery (mating with males at 8 weeks of age). A: control group, B: DecaBDE-treated group

Forty-eight female SD rats (Figure 1) were allowed one week to acclimate prior to treatment and were subsequently grouped into two weight-matched groups: the Control group and DecaBDE-treated group. Then, rats in the DecaBDE-treated group were fed low-dose (1000 mg/kg body weight (bw) per day) DecaBDE (CAS No. 1163-19-5, purity: >98%, Sigma Aldrich, St. Louis, MO, USA) using a modified version of the method developed by Miyaso et al. [27,28] from 5 weeks of age to delivery. 

The female rats were mated separately with 24 untreated male rats at 8 weeks of age. Vaginal smears were subsequently examined for sperm. This day was considered day 0 of gestation (GD0) if sperm were detected with a microscope. Six pregnant rats in each group were separately sacrificed on GD7, GD13, and GD19. Any abnormal reproductive appearance of pregnant rats was recorded. The number of fetuses in each pregnant rat on GD19 and the number of pups in each litter on postnatal day 1 (PND1) were also recorded. Two newborn pups were randomly chosen from each litter before reaching one day of age. The weights of newborn pups were also measured.

#### 2.2.3. Data Analysis

Normality of raw data was tested using the Shapiro-Wilk test. *t*-tests were used to determine the differences in the weight and length of infants between the two groups. Categorical variables were analyzed by the chi-square test. *p* < 0.05 was considered significant. Analyses in our study were completed using the Stata SE12.0 software.

## 3. Results

### 3.1. Analysis of the Results of Human Studies

A total of 102 potential articles were initially searched, and 38 duplicate articles were excluded. After reviewing the abstracts of the remaining articles, 51 articles were excluded because they were irrelevant. Eight articles were excluded due to the lack of data for analysis after full-text review. Finally, five published articles met the inclusion criteria. The flow of the literature search and selection process is shown in Figure S1. Seven studies were finally analyzed in our study because two articles included two studies. The characteristics of the studies included in our study are summarized in Table 1. All included individuals were recruited from cohort studies that were designed to analyze the correlation between PEP and IBO.

#### 3.1.1. The Correlation between PEP and Birth Weight

Seven studies provided necessary data for the analysis of the relationship between PEP (ng/g of lipids) and birth weight (g) (Figure S2). A significant negative relationship was found between human PBDE burden and birth weight using a random-effects model (β = −50.598; 95% CI −95.914, −5.282; *I*^2^ = 11.8%; *p* < 0.05). The seven studies were then stratified by gender. Subgroup one was composed of two studies on the correlation between PEP and birth weight of female infants. No relationships were found between PEP and birth weight (β = 37.766; 95% CI −81.425, 156.957; *I*^2^ = 0%; *p* > 0.05). Subgroup two comprised two studies on the correlation between PEP and birth weight of male infants. PEP was negatively correlated with the birth weight of male infants (β = −121.456; 95% CI −230.139, −12.773; *I*^2^ = 0%; *p* < 0.05). Subgroup three comprised three studies on this correlation regardless of gender. No relationships were found between PEP and birth weight (β = −54.388; 95% CI −115.982, 7.206; *I*^2^ = 33.4%; *p* > 0.05). The results using the two models (fixed-effects and random-effects models) were consistent with each other. There was no obvious publication bias in our analysis according to Egger’s test and Begg’s test (*p* = 1.00, 0.846, respectively) (Figure S3).

We further extracted data from four included studies and performed separate analyses for the correlation between single PBDE congener and IBO (BDE-47, BDE-99, BDE-100 and BDE-153). Although no statistically significant associations were found, we observed a trend of negative associations between each congener and IBO using a random-effects model (Figures S4–S7).

#### 3.1.2. The Correlation between PEP and Other Birth Outcomes

Three studies provided data for the analysis of the relationship between PEP (ng/g of lipids) and birth length (cm). No relationships were found between PEP and birth length (β = −0.33; 95% CI −0.74, 0.07; *I*^2^ = 38.8%; *p* = 0.107) (Figure S8). Three studies provided data for the analysis of the relationship between PEP (ng/g lipids) and head circumference (cm). There were no relationships between PEP and head circumference (β = −0.175; 95% CI −0.418, 0.069; *I*^2^ = 30.4%; *p* = 0.16) (Figure S9). Subgroup analysis, sensitivity analysis, and publication bias analysis were not performed because there were not enough studies.

### 3.2. Experimental Animal Study

#### 3.2.1. The Effect of PEP on the Number of Embryos or Fetuses during Pregnancy and Pups after Delivery

**Figure 2:**
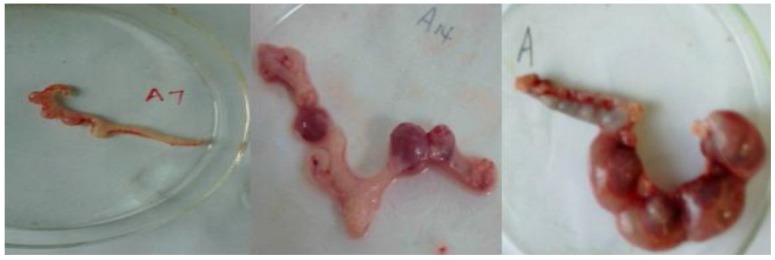
The abnormal presence of three uteri in pregnant rats with less than 4 offspring in the DecaBDE-treated group.

Thirty-six pregnant rats were used to observe the effect of PEP during pregnancy. Three abnormal pregnant rats with an offspring number below five were observed in the DecaBDE-treated group (Figure 2), and none were observed in the control group. However, there was no significant difference in the number of abnormal pregnant rats between the control group and DecaBDE-treated group (*p* = 0.229).

**Figure 3:**
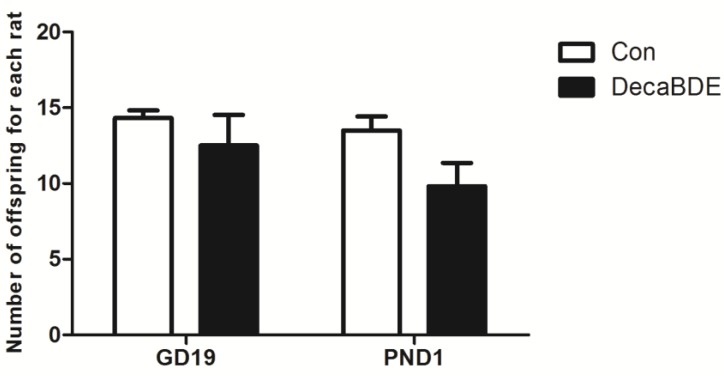
The number of fetuses on gestation day 19 in each rat and the number of pups in each litter on postnatal day 1 in the control (Con) group and DecaBDE-treated (DecaBDE) group. GD19: gestational day 19; PND1: postnatal day 1. *p* = 0.44, 0.32, respectively.

There was no difference in the number of fetuses in each rat on GD19 or in the number of pups in each litter on PND1 between the DecaBDE-treated and control groups (Figure 3).

#### 3.2.2. The Effect of PEP on the Birth Outcomes of Newborn Pups

**Figure 4:**
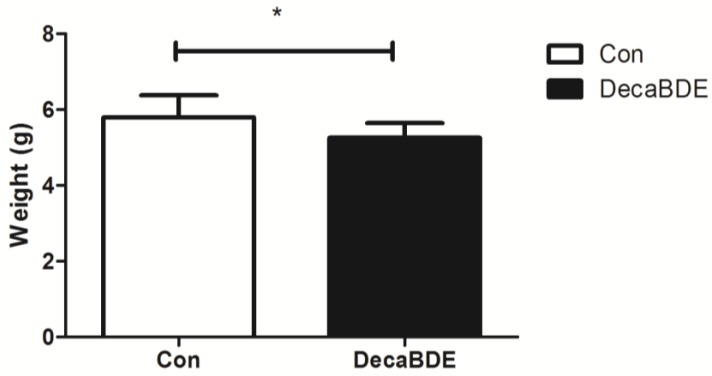
Comparison of the birth weight of newborn pups between the control group and DecaBDE-treated group; *: *p* = 0.0132.

The body weight of newborn pups in the experimental group was significantly (5.26 ± 0.39 vs. 5.8 ± 0.58, *p* = 0.0132) lower than that of those in the control group (Figure 4). The median birth weight of 24 newborn pups was 5.56 g. The number (*n* = 10) of pups weighing less than 5.56 g in the DecaBDE-treated group was greater than that (*n* = 2) in the control group (*p* = 0.003).

## 4. Discussion

Several epidemiological studies analyzed the correlation between PEP and birth weight. However, the results were inconsistent. Some studies showed significantly negative associations between PBDEs and birth weight, while other studies showed positive or negative associations that were not statistically significant. Currently, there is still no convincing conclusive evidence regarding this debate in human studies. Recently, concerns about DecaBDE have increased because its concentration is increasing in the environment. As the only PBDE congener still used widely today, DecaBDE could be metabolically debrominated to lower brominated congeners in vivo [29,30]. Thus, the impacts of PEP and IBO were predominantly due to high-molecular weight DecaBDE, either directly or indirectly. Levels of PBDE congeners in house dust with similar degrees of bromination were significantly positive intercorrelated [31]. Several epidemiological studies [32,33] showed that levels of 2,2′,4,4′-Tetrabromodiphenyl ether (BDE-47), 2,2′,4,4′,5-Pentabromodiphenyl ether (BDE-99), 2,2′,4,4′,6-Pentabromodiphenyl ether (BDE-100), 2,2′,4,4′,5,5′-Hexabromodiphenyl ether (BDE-153), 2,2′,3,4,4′,5,6-heptabromodiphenyl ether (BDE-183) and DecaBDE (BDE-209) in humans were positively correlated with each other. Humans were exposed to multiple congeners, such as pentabromodiphenyl ether (PentaBDE), octabromodiphenyl ether (OctaBDE) and DecaBDE [12,14], but only BDE-47, BDE-99, BDE-100, and BDE-153 were usually measured to explore the relationship between PBDE burden and birth outcomes in human studies. A few epidemiological studies [11,34,35,36] measured the levels of DecaBDE, and the levels of DecaBDE in these studies comprised a considerable proportion of the PBDEs. As a pollutant source of PBDEs, DecaBDE should be used as a target congener in the meta-analysis, which is a rational but infeasible method to explore the correlation between DecaBDE and IBO because eligible epidemiological studies evaluating this correlation were rare. We evaluated the relationship between total level of major congeners and IBO in our meta-analysis. Levels of PBDE congeners were positively correlated with each other [32,33]. We proposed that the relationship present in our meta-analysis may be consistent with that between DecaBDE and IBO. In one animal study [37], rats were exposed to DecaBDE over three months. Compared to the control group, the DecaBDE-exposed rats had significantly elevated levels of 9 PBDE congeners, such as BDE-183, OctaBDE (BDE-196, BDE-197, BDE-202, BDE-203), and BDE-209, in the tissues. In our experimental study, DecaBDE was considered an optimal congener for evaluating the correlation between PEP and birth outcomes.

To our knowledge, this is the first study to comprehensively assess the association between PEP and birth weight and to suggest that gender difference may be a heterogeneous factor affecting the association. Two of the included studies were statistically significant, while the other five were not. Without our analysis, it may be mistakenly assumed that there was no relationship between them. The conflicting results were due to the fact that the sample size in some epidemiological studies was not large enough to achieve statistical significance. By pooling all the results, we found that PEP was significantly related to the decreased birth weight of offspring. However, whether something else, positively related to serum PBDEs levels, cause adverse effect on birth weight directly or indirectly is still unknown. So, evidences from observational epidemiological studies were unsuitable to conclude the cause and effect relationship between PEP and lower birth weight. It requires further to be verified by experimental animal studies. In our animal study, rats exposed to DecaBDE also have lower birth weight, which indicate that PEP may be a causal factor in offspring lower birth weight.

Although the relationship between DecaBDE and IBO was not evaluated in our meta-analysis, a study performed by Chao et al. [11] found a significant negative association between infant birth weight and DecaBDE as well as BDE-47, BDE-99 and BDE-100 in breast milk individually. We further extracted data from four included articles and performed separate analyses for the correlation between single PBDE congeners and IBO. We observed similar trends of negative associations between each congener and IBO.

Subsequently, we stratified the seven human studies by gender and found that compared with female infants, male infants may be more predisposed to the adverse birth outcomes of PBDE exposure. This finding was in accordance with another animal experiment performed previously by Kim et al. [17]. Different responses to PBDE congeners between genders were also found in other animal studies [17,38,39]. These findings should be verified in further comprehensive studies.

There are several limitations in our study: (1) The included epidemiological studies were not sufficient for further analysis of factors affecting the correlation between PEP and birth outcomes in these human studies. Race, age, median of five PBDE congeners, gender and other variables were assessed to identify the factors affecting the correlation. Finally, we observed that gender difference may be a heterogeneous factor. Although there were only two studies in subgroup 1 and subgroup 2, the results of this subgroup analysis will be helpful for improving the protocols of future studies on this topic. The subgroup analysis indicated that the heterogeneity of β values in subgroup 3 (both male and female) were notably higher than those of subgroup 1 (female) and 2 (male). These findings suggested that gender may be a heterogeneous factor affecting the association; (2) Gender differences were not verified in the animal study because the experimental animal study had been finished prior to completion of the subgroup analysis for gender. Initially, our animal study was designed to verify the results of the meta-analysis regarding whether PEP adversely impacts birth, and we did not focus on the importance of gender until the subgroup analysis of gender difference was completed. The loss was irretrievable because all pups had been sacrificed at that time. Thus, the results about gender difference should be interpreted with caution; (3) Meta-regression was not considered because the number of studies in our meta-analysis is less than 10; (4) Only published English language studies were included in our meta-analysis; publication and selection bias may be present despite a comprehensive search; (5) Because levels of PBDE congeners are intercorrelated, the significant associations between PBDEs and IBO may be derived from the effect of a single congener. However, the result of our study may be helpful to limit the production and utility of DecaBDE, and the level of the single congener will decrease accordingly; (6) The mechanism of the relationship was not investigated in our experimental animal study. The potential mechanisms responsible for the correlation between PBDE exposure and developmental toxicity have not been fully elucidated. The potential mechanisms responsible for the correlation between PBDE exposure and developmental toxicity have not been fully elucidated.

Experimental evidence suggests that PBDEs in maternal blood can partially cross the placenta [40,41,42] and disrupt thyroid function [17,43,44]. Thyroid disruption has been considered a crucial pathway linking PEP and IBO. The development of fetuses depends on the thyroid hormone levels of the mother during pregnancy (completely during the first half and partially during the second half); therefore, the development of fetuses may be negatively impacted by disruption of the thyroid hormone supply from reaching the fetus. Placental deiodinases (Dio3) play important roles in mediating the levels of thyroid hormones in fetuses [45]. Several studies [46,47] have demonstrated that OH-BDEs and PBDEs can significantly inhibit the activity of thyroid hormone deiodinase. Therefore, PBDEs that inhibit the activity of Dio3 in the placenta may be a crucial factor in adverse birth outcomes.

We also found that there was a gender difference in birth weight in the responses to prenatal exposure to PBDEs in the subgroup analysis of epidemiological studies. Different responses of exposure to PBDE congeners between genders were also found in other animal studies [17,38,39]. The findings should be verified in further comprehensive studies.

## 5. Conclusions

Our study contributes to the growing evidence of the deleterious effect of PEP on birth outcomes. The findings suggested that gender differences may be the most important factor affecting this relationship. This conclusion requires further evidence, namely, a prospective study with a large sample size.

## Figures and Tables

**Table 1:** Abstracted major information from the included epidemiological studies on the relationship between polybrominated diphenyl ether (PBDE) levels and birth outcomes.

Author	Year	Country	Median Serum PBDE ^b^	Sample Size	PBDE Congeners	Gender of Infants	Effect Size β (95% CI) ^c^
Harley	2011	USA Mexico	22.9	286	47, 99, 100, 153	M, F	−140.2 * (−254.1, −26.3)
Lignell	2013	Sweden	1.67 ^a^	185	47, 99, 100, 153	M	−126 * (−247.52, −4.48)
Lignell	2013	Sweden	1.67 ^a^	161	47, 99, 100, 153	F	32 (−105.2, 169.2)
Miranda	2015	USA	34.69	136	47, 99, 100, 153	M, F	−31.9 (−89.2, 25.41)
Chen	2015	China	12.84	104	47, 99, 100, 153, 28	F	55.51 (−185.17, 296.19)
Chen	2015	China	12.84	111	47, 99, 100, 153, 28	M	−103.29 (−346.254, 139.69)
Serme-Gbedo	2016	Canada	0.19	349	47, 99, 100, 153	M, F	−25.4 (−128.7, 77.9)

Note: *: *p* < 0.05; M: male; F: female; ^a^: estimated value; ^b^: Sum of 2,2′,4,4′-Tetrabromodiphenyl ether (BDE-47), 2,2′,4,4′,5-Pentabromodiphenyl ether (BDE-99), 2,2′,4,4′,6-Pentabromodiphenyl ether (BDE-100), and 2,2′,4,4′,5,5′-Hexabromodiphenyl ether (BDE-153) (ng/g lipid); ^c^: β values for the correlations between log PBDEs and birth weight (g); CI: Confidence Interval.

## Supplementary Materials

The following are available online at www.mdpi.com/1660-4601/14/3/268/s1. Figure S1: Flow diagram of the article selection process; Figure S2: Forest plot of pooled β values for the association between prenatal exposure to PBDEs and birth weight; Figure S3: The Begg’s funnel plot of the publication bias (*p* = 0.846); Figure S4: Forest plot of pooled β values for the association between prenatal exposure to BDE47 and birth weight; Figure S5: Forest plot of pooled β values for the association between prenatal exposure to BDE99 and birth weight; Figure S6: Forest plot of pooled β values for the association between prenatal exposure to BDE100 and birth weight; Figure S7: Forest plot of pooled β values for the association between prenatal exposure to BDE153 and birth weight; Figure S8: Forest plot of pooled β values for the association between prenatal exposure to PBDE s and birth length; Figure S9: Forest plot of pooled β values for the association between prenatal exposure to PBDEs and head circumference.
