# Supplementary material for: Correlation between Prenatal Exposure to Polybrominated Diphenyl Ethers (PBDEs) and Infant Birth Outcomes: A Meta-Analysis and an Experimental Study
Source: Int J Environ Res Public Health. 2017 Mar 7;14(3):268. doi: 10.3390/ijerph14030268 (PMC5369104; doi:10.3390/ijerph14030268)
Supplement: Supplementary file 1 [file ijerph-14-00268-s001.pdf]

Supplementary Materials: Correlation between Prenatal Exposure to Polybrominated Diphenyl Ethers (PBDEs) and Infant Birth Outcomes: A Meta-Analysis and An Experimental Study

Xuemin Zhao, Shiqiao Peng, Yang Xiang, Yali Yang, Jing Li, Zhongyan Shan and Weiping Teng

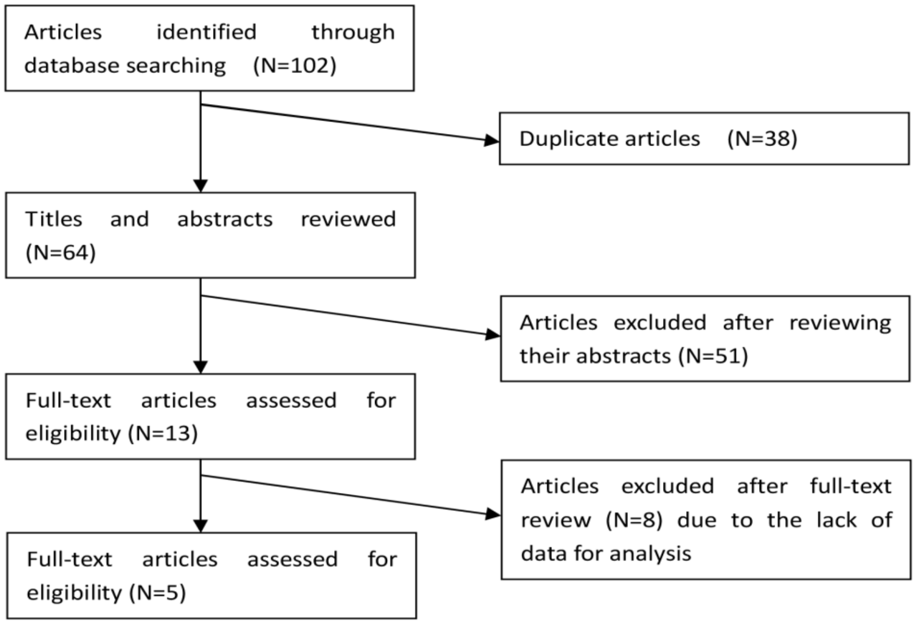

Figure S1. Flow diagram of the article selection process.

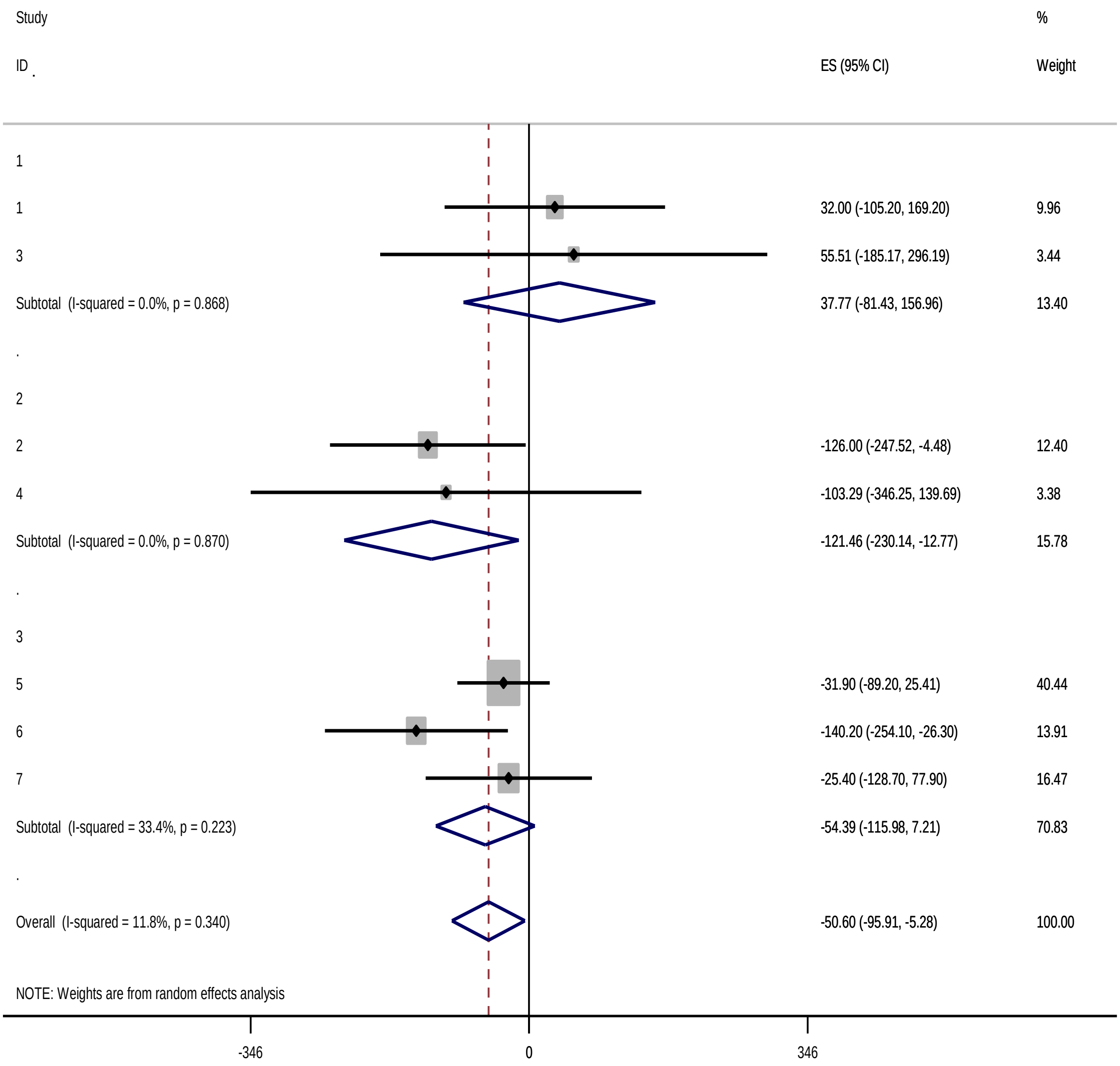

Figure S2. Forest plot of pooled β values for the association between prenatal exposure to polybrominated diphenyl ethers (PBDEs) and birth weight. ES: effect size; CI: confidence interval; ID: identification; I-squared: I^2

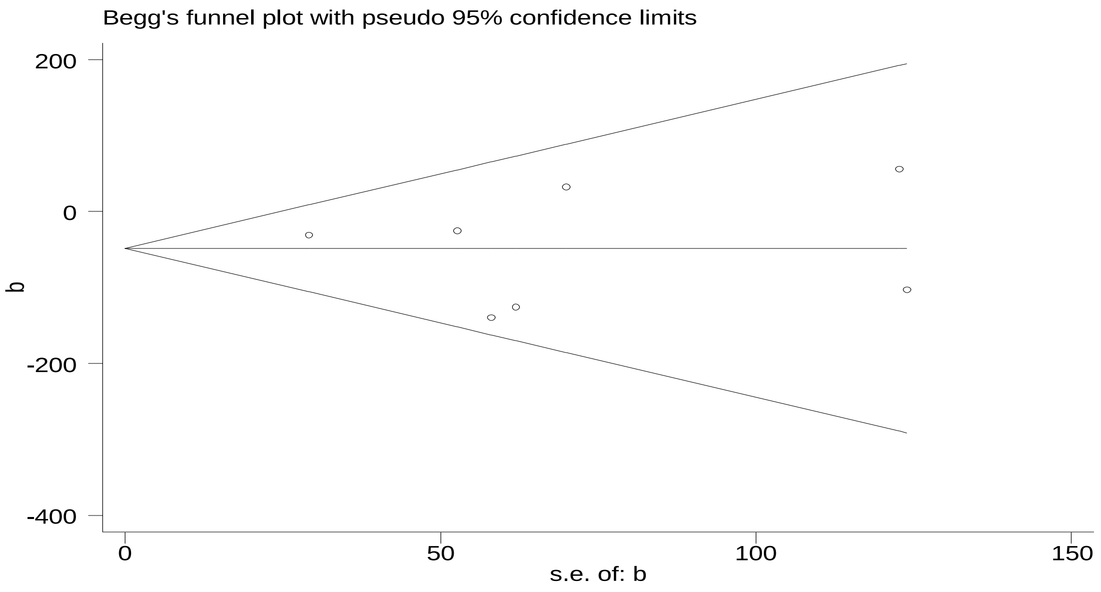

Figure S3. The Begg’s funnel plot of the publication bias ($p = 0.846$).

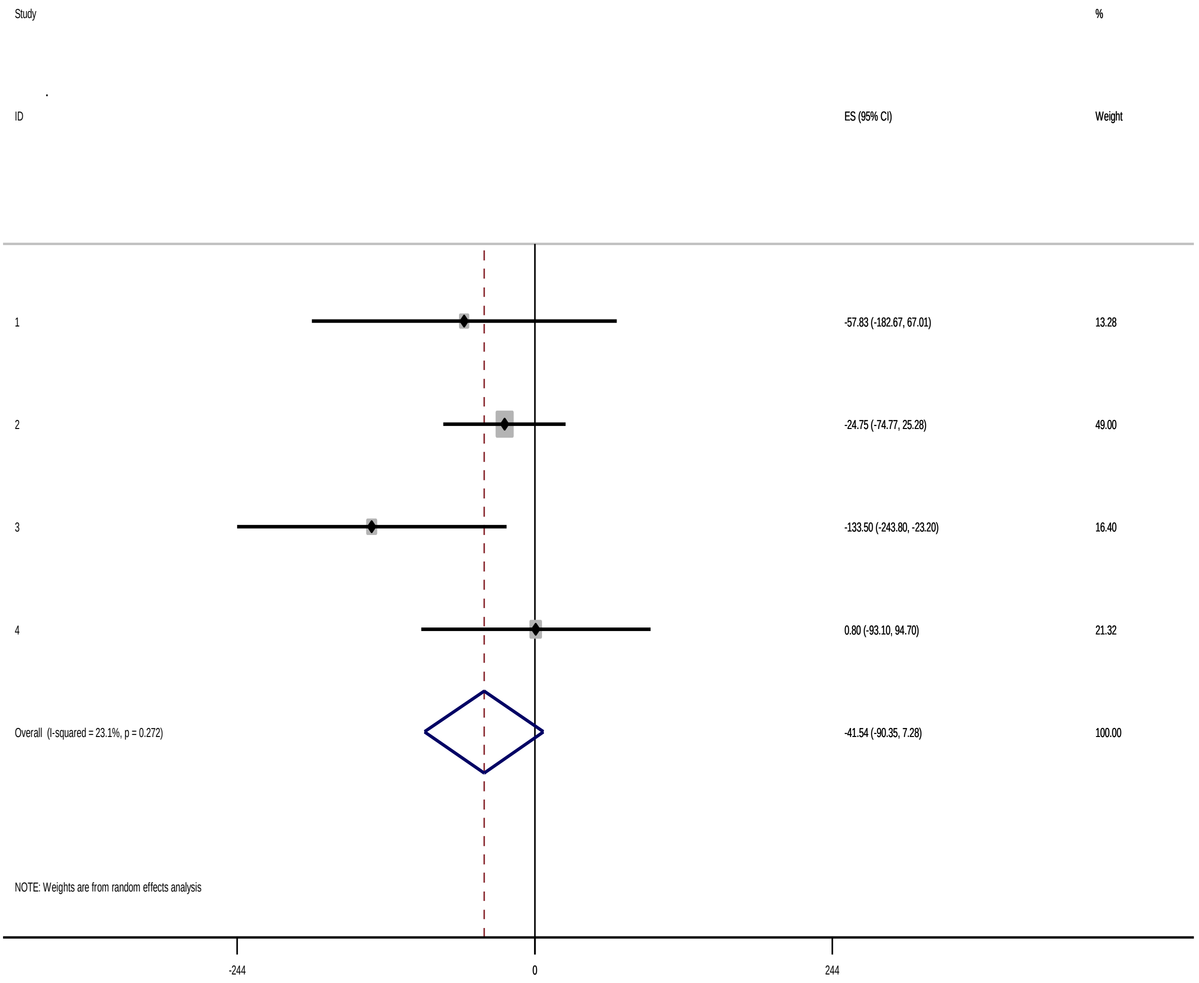

Figure S4. Forest plot of pooled β values for the association between prenatal exposure to 2,2',4,4'-Tetrabromodiphenyl ether (BDE47) and birth weight.

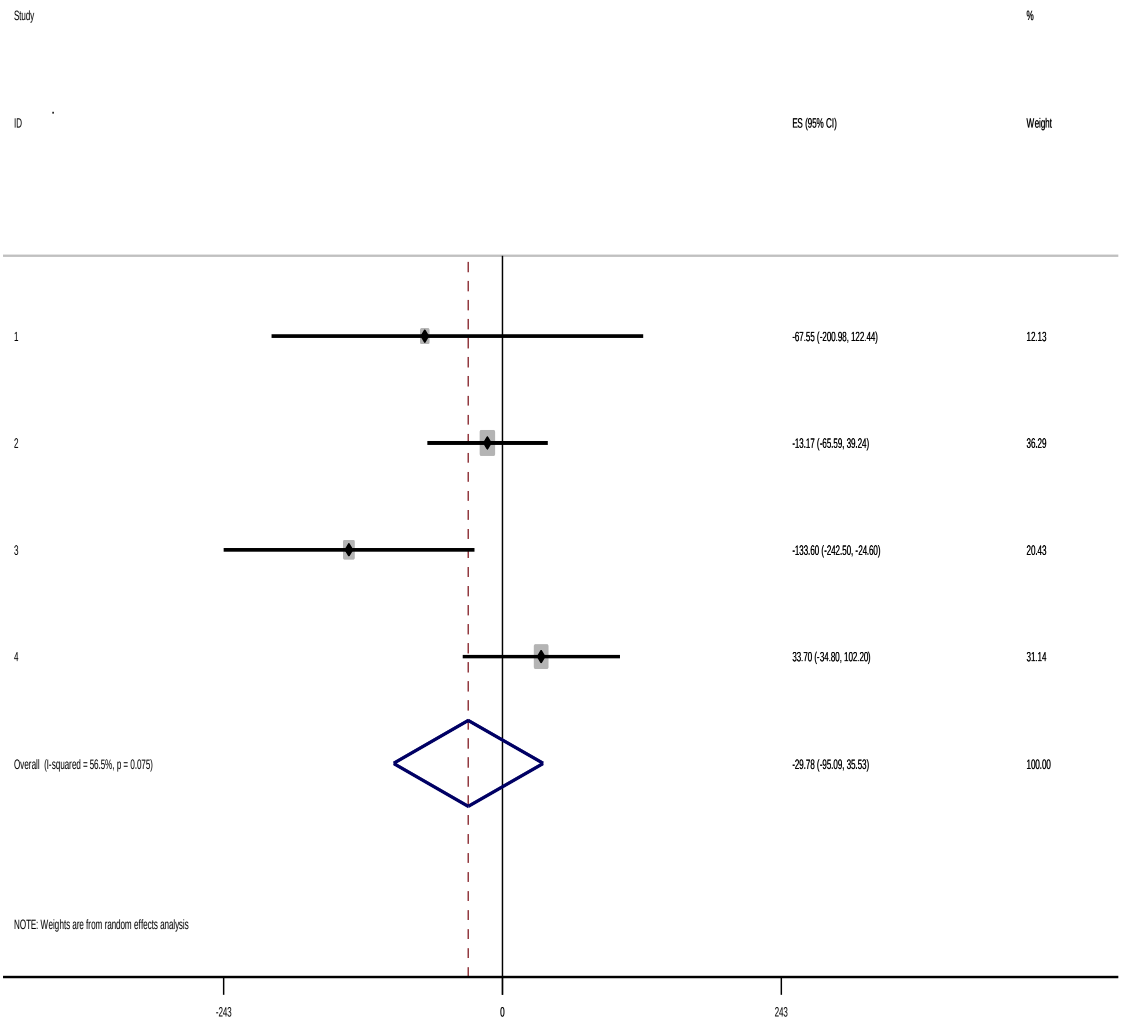

Figure S5. Forest plot of pooled β values for the association between prenatal exposure to 2,2',4,4',5-Pentabromodiphenyl ether (BDE99) and birth weight.

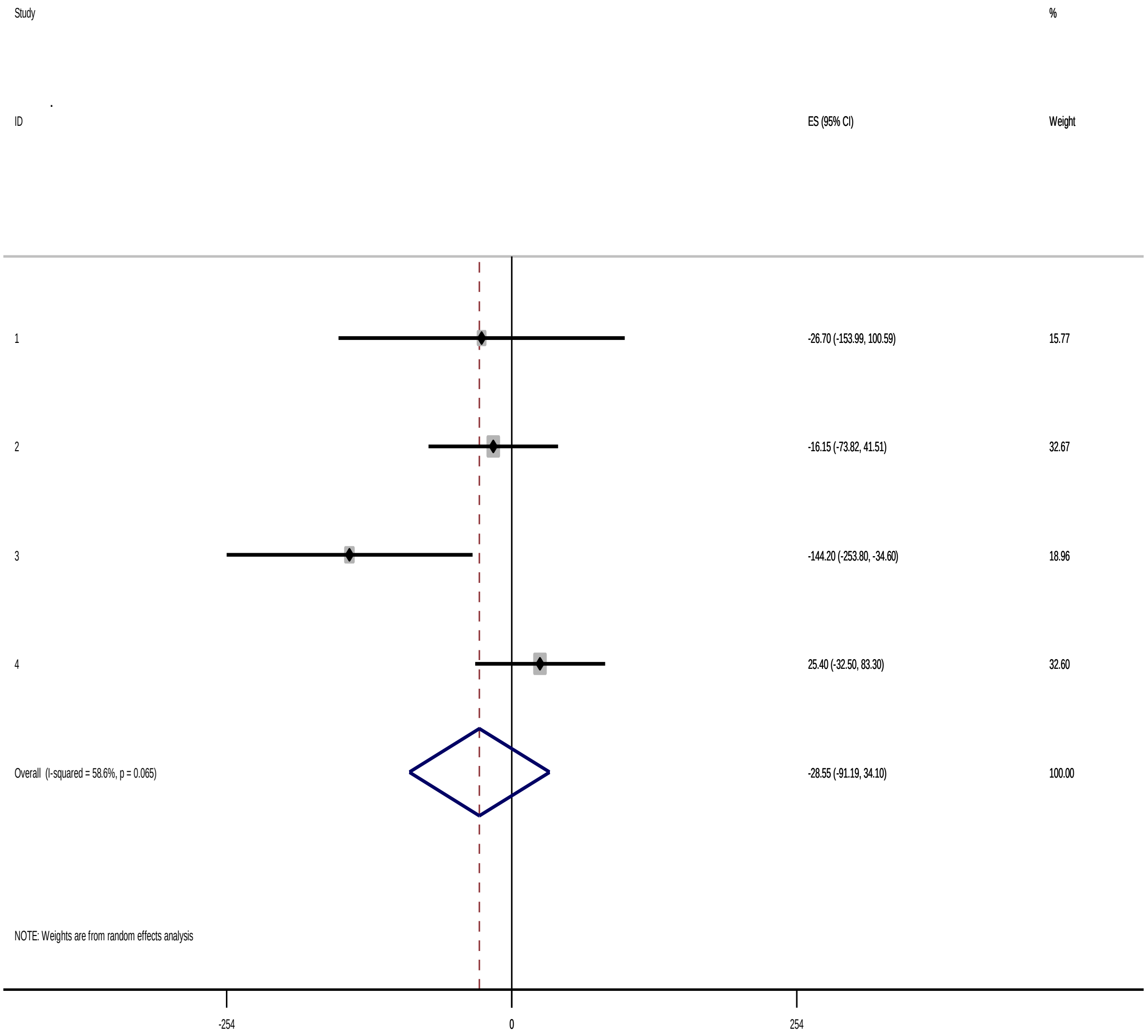

Figure S6. Forest plot of pooled β values for the association between prenatal exposure to 2,2',4,4',6-Pentabromodiphenyl ether (BDE100) and birth weight.

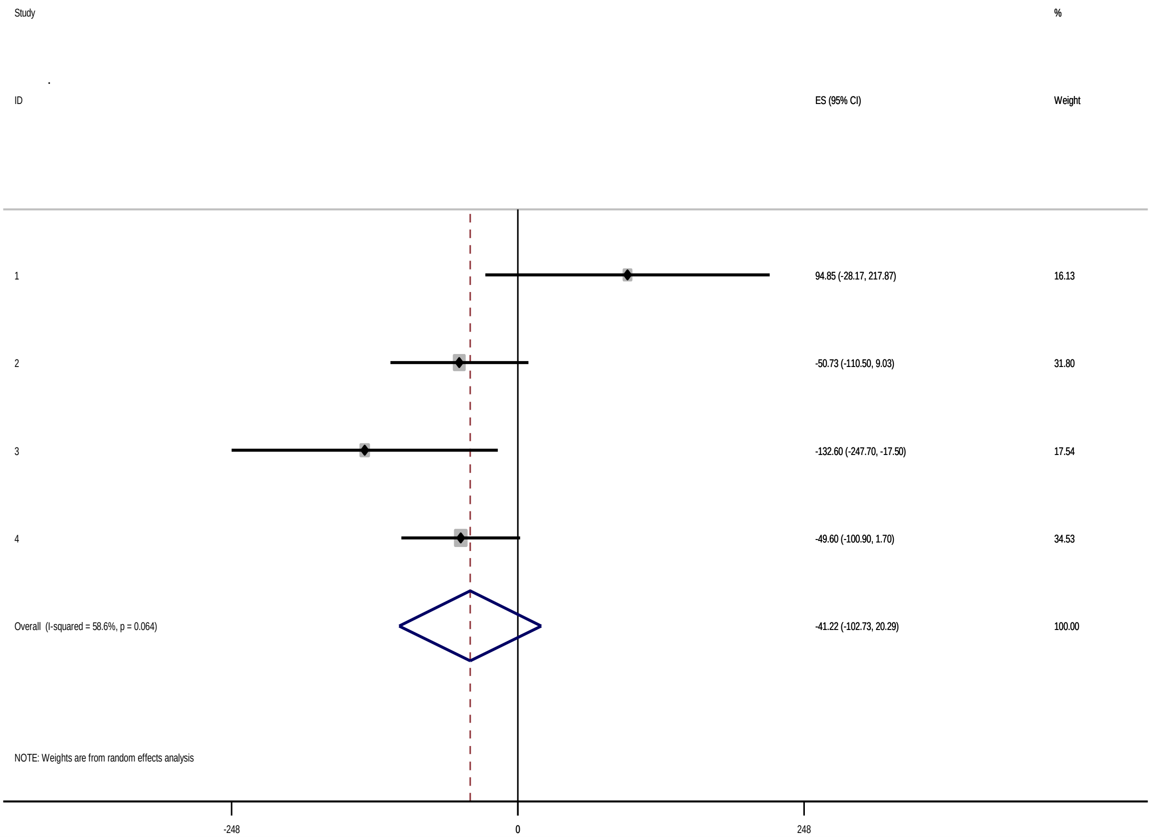

Figure S7. Forest plot of pooled β values for the association between prenatal exposure to 2,2′,4,4′,5,5′-Hexabromodiphenyl ether （BDE153） and birth weight.

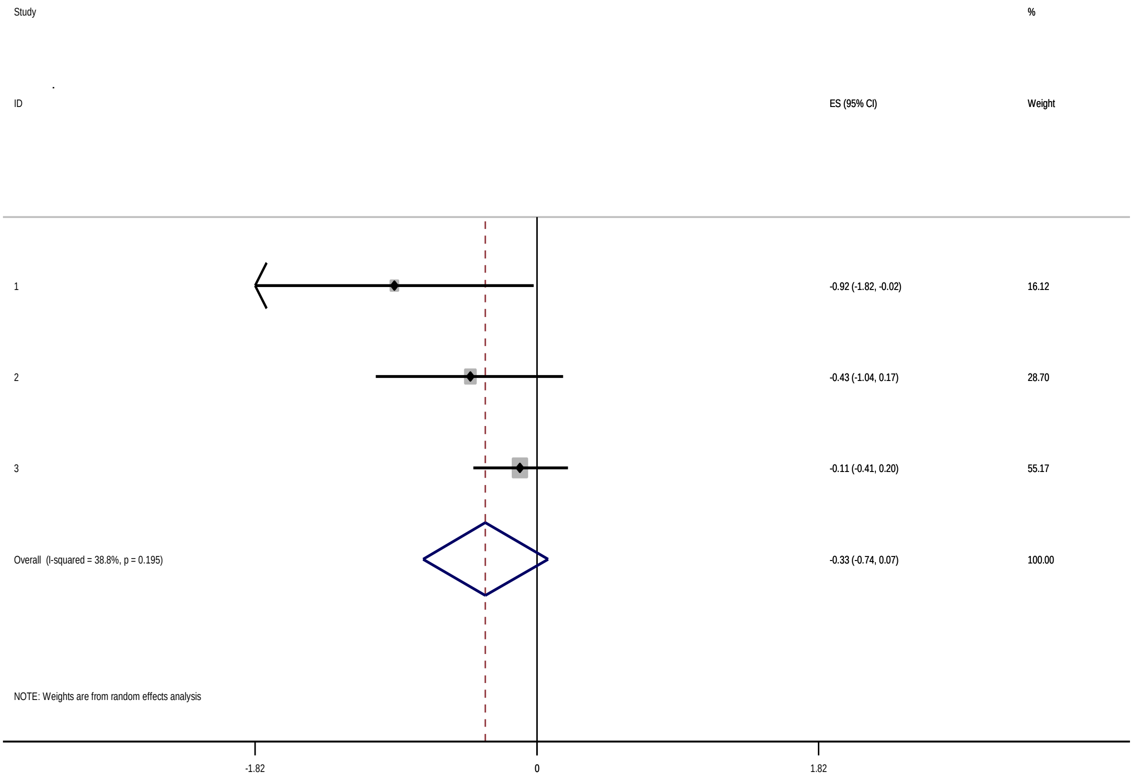

Figure S8. Forest plot of pooled β values for the association between prenatal exposure to PBDEs and birth length.

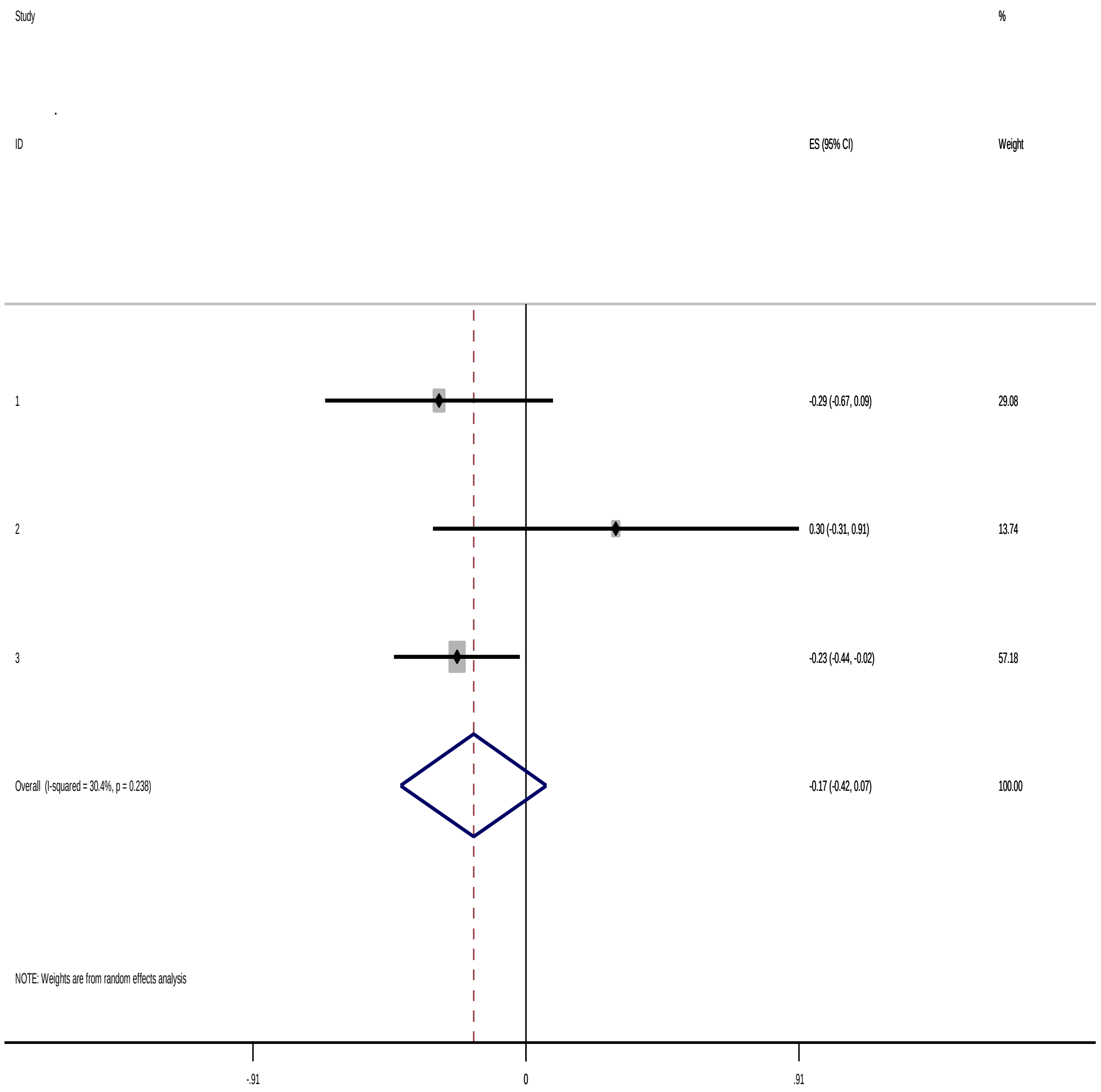

Figure S9. Forest plot of pooled β values for the association between prenatal exposure to PBDEs and head circumference.
